# Increased serum albumin corrected anion gap levels are associated with poor prognosis in sepsis-induced coagulopathy patients

**DOI:** 10.1371/journal.pone.0347039

**Published:** 2026-04-16

**Authors:** ZhenHong Jiang, ShiJin Lv, ZengYan Fu, GuoHu Zhang

**Affiliations:** Department of Emergency, The Affiliated Hospital of HangZhou Normal University, HangZhou, Zhejiang, China; King Fahd Military Medical Complex, SAUDI ARABIA

## Abstract

**Background:**

Sepsis-induced coagulopathy (SIC) is associated with high mortality, and acid-base disturbances are common in critically ill patients with this condition. The anion gap (AG) is affected by serum albumin levels, suggesting that the albumin-corrected anion gap (ACAG) may serve as a more accurate prognostic marker. However, the relationship between ACAG and outcomes in SIC remains unclear.

**Methods:**

This retrospective cohort study utilized data from the Medical Information Mart for Intensive Care IV (MIMIC-IV) database (2008–2019). Adult patients (≥18 years) diagnosed with SIC within 24 hours of ICU admission were enrolled. SIC was defined according to the International Society on Thrombosis and Haemostasis (ISTH) criteria combined with Sequential Organ Failure Assessment (SOFA) scores. ACAG was calculated as: ACAG = AG + 2.5 × (4.4 – Albumin [g/dL]). The optimal cutoff value (17.7 mmol/L) was determined using X-tile software. The primary outcome was in-hospital 30-day all-cause mortality. Time-dependent Cox proportional hazards models, Kaplan-Meier (KM) analysis, and receiver operating characteristic (ROC) curves were performed. Subgroup and interaction analyses were conducted to assess effect modification by malignancy status.

**Results:**

A total of 3,846 patients were included (mean age 64.8 years; 38.4% female). Non-survivors exhibited significantly higher ACAG levels than survivors (median [IQR]: 21.4 [18.5–24.8] vs. 17.6 [15.2–20.3] mmol/L, *P* < 0.001). KM analysis showed that high ACAG (≥17.7 mmol/L) was associated with significantly lower in-hospital 30-day survival (log-rank *P* < 0.001). In the time-dependent Cox model (adjusted for SOFA, SAPS II, and lactate), high ACAG was independently associated with increased mortality (HR = 3.04, 95% CI: 1.88–4.91, *P* < 0.001). ACAG demonstrated superior discrimination compared to uncorrected AG (AUC: 0.633 vs. 0.620, DeLong test *P* < 0.001). Notably, a significant interaction was observed between ACAG and malignancy (*P* for interaction < 0.001), with ACAG showing stronger predictive value in non-malignant patients (HR = 3.60, *P* < 0.001) than in those with cancer (HR = 1.59, *P* = 0.477).

**Conclusions:**

Elevated ACAG is independently associated with increased in-hospital 30-day mortality in SIC patients and outperforms uncorrected AG. Its calculation requires no additional cost or testing, making it a practical bedside risk stratification tool, particularly for non-malignant patients.

## Introduction

Sepsis is a life-threatening condition triggered by severe infections. In sepsis, dysregulated inflammation can lead to life-threatening tissue damage, shock, and multiple organ dysfunction syndrome (MODS). Sepsis-induced coagulopathy (SIC) is a critical complication of sepsis [1]. Characterized by endothelial injury and coagulation disorders, SIC significantly increases mortality risk in patients with sepsis [1,2]. The intricate crosstalk between the inflammatory and coagulation systems plays a pivotal role in sepsis pathogenesis [3,4]. During sepsis, the body activates the coagulation system as a defense mechanism against pathogen invasion [3]. On one hand, uncontrolled leukocyte activation damages vascular endothelial cells and initiates coagulation, leading to extensive thrombosis [5]. On the other hand, numerous immunothrombi form within blood vessels. Immunothrombosis—an intravascular antibacterial defense mechanism—involves local microvascular thrombus formation, fibrin generation, and recruitment of immune cells and platelets [3]. Additionally, impaired physiological anticoagulant pathways, excessive coagulation activation, and fibrinolysis inhibition may cause widespread microcirculatory thrombosis, ultimately resulting in isolated organ damage or MODS [3].

Acid-base imbalance is prevalent among inpatients in the intensive care unit (ICU) and is closely associated with the mortality of various diseases [6]. It is well established that the anion gap (AG), which reflects the concentration difference between unmeasured serum anions and cations, is one of the most commonly used biomarkers for diagnosing acid-base imbalance and identifying metabolic acidosis [7]. AG is primarily composed of albumin (ALB), lactate, sulfate, phosphate, and other unmeasured ions [7]. However, due to the net negative charge of ALB, alterations in its concentration may significantly impact AG levels [8]. Consequently, an increasing number of researchers have proposed that the albumin-corrected anion gap (ACAG) is more suitable for assessing acid-base balance disorders and evaluating disease progression [9]. Previous studies have indicated that elevated ACAG levels are strongly correlated with adverse clinical outcomes in patients with acute myocardial infarction, acute kidney injury (AKI), acute pancreatitis, and asthma [7,9–11].

The association between ACAG and SIC remains to be elucidated. In view of the intricate crosstalk among inflammation, coagulation, and acid-base balance in SIC, and considering that ACAG not only reflects the acid-base state but may also be affected by the pathophysiological processes of sepsis, it is reasonable to postulate that ACAG may be associated with the prognosis of SIC [12]. Therefore, by extracting clinical hospitalization data of SIC patients from the Medical Information Mart for Intensive Care IV (MIMIC-IV) database between 2008 and 2019, we aimed to investigate the correlation between ACAG and in-hospital 30-day mortality in these patients. Moreover, we also aimed to evaluate the predictive value of ACAG for SIC prognosis.

## Materials and methods

### Data source

The MIMIC-IV database, a publicly accessible **critical care research resource**, provides de-identified clinical data from the intensive care units of Beth Israel Deaconess Medical Center (BIDMC) (https://physionet.org/content/mimiciv/2.2/). Following successful completion of the required CITI Data or Specimens Only Research training course and examination, one author obtained authorized access to the database and was responsible for data extraction (certification number: 60409192). Given that the database has undergone formal de-identification in accordance with HIPAA Safe Harbor provisions, additional ethical approval was deemed unnecessary [13].

### Study population

Within the framework of this study, we implemented rigorous inclusion criteria. Initially, the patients incorporated into our study were those with SIC during their hospital admission spanning from 2008 to 2019. The diagnostic criteria adhered to were those of the International Sepsis Definitions for Sepsis and Organ Dysfunction (ISFD) [12]. The diagnosis was established when there was a reduction in platelet count to 100–150 × 10⁹/L, which equates to a score of 1; when the platelet count is less than 100 × 10⁹/L, a score of 2 is assigned; prolonged prothrombin time/international normalized ratio (PT-INR) between 1.2 and 1.4 scores 1 point, and when it exceeds 1.4, a score of 2 points is given; additionally, the Sequential Organ Failure Assessment (SOFA) score, which is calculated from the sum of respiratory, hepatic, cardiovascular, and renal dysfunction scores, is also considered: 1 point is assigned for a score of 1, and scores of 2 or higher are assigned for scores of 2 or more. A total score reaching or exceeding 4 points results in the diagnosis of SIC [14,15]. Secondly, we limited inclusion to patients aged ≥18 years. Finally, we took into account only those patients for whom albumin and anion gap measurements were obtainable within 24 hours of their ICU admission. When the same patient had multiple admissions, we incorporated solely the information from the first admission and the initial ICU stay. The establishment of these inclusion criteria aimed to guarantee the accuracy and reliability of the results of our study.

### Data extraction

In this research, we retrieved demographic data, laboratory parameters, illness severity scores, comorbidities, and life-sustaining interventions from the MIMIC-IV database via Structured Query Language. The laboratory measurements and life-sustaining treatments we extracted were confined to the first 24 hours after the patients’ admission to the intensive care unit.The demographic features covered age, gender, and ethnicity. Regarding comorbidities, they included diabetes, liver disorder, myocardial infarction, congestive heart failure, chronic obstructive pulmonary disease (COPD), renal disease, malignant cancer, and hypertension. Laboratory indices consisted of white blood cell count, hemoglobin level, platelet count, aspartate aminotransferase (AST), alkaline phosphatase (ALP), alanine aminotransferase (ALT), total bilirubin, anion gap, international normalized ratio (INR), albumin, blood urea nitrogen (BUN), lactate, glucose, prothrombin time (PT), and partial thromboplastin time (PTT). The life-sustaining interventions comprised vasopressor use, renal replacement treatment (RRT), and invasive mechanical ventilation (IMV). Scoring systems: Simplified Acute Physiology Score II (SAPS II), SIC score, and SOFA score. ACAG was computed as ACAG = AG + 2.5 × (4.4 – Albumin [g/dL]) [16,17].

### Outcomes

The primary study outcome was in-hospital 30-day mortality, with the time origin standardized to the date of ICU admission. Two key temporal definitions were applied: (1) ICU length of stay (LOS) was defined as the total duration of the patient’s ICU hospitalization; (2) the outcome observation window was fixed at 30 days post-ICU admission. The primary outcome was in-hospital mortality within 30 days of ICU admission. Only deaths occurring during the index hospitalization were counted. At the 30-day follow-up endpoint, patients were dichotomized into two groups (survivors vs. non-survivors) based on their confirmed survival status at this time point.

### Groups

We used X-tile software to identify the optimal cutoff value of ACAG (17.7 mmol/L), stratifying patients into normal and high ACAG groups with in-hospital 30-day mortality as the primary endpoint. Subsequent validation via Brier score, bootstrap-based calibration plot (500 resamples), and decision curve analysis (DCA) confirmed the cutoff’s robust reliability (S1 Fig and S2 Fig): the categorical ACAG model yielded a Brier score of 0.1966 (95% CI: 0.1891–0.2059), indicating low prediction error; the observed in-hospital 30-day mortality probabilities of both ACAG subgroups closely aligned with the ideal calibration line (all 95% CIs covered the reference line), reflecting well-calibrated risk stratification; and DCA results demonstrated that the categorical model provided substantial net benefit across a wide range of threshold probabilities, collectively confirming that the 17.7 mmol/L cutoff of ACAG is both statistically reliable and clinically useful for risk stratification of SIC patients.

### Statistical analysis

We used the Kolmogorov–Smirnov test to check for normality in continuous variables. For normally distributed data, we reported results as mean (SD) and used the Student’s t-test for group comparisons; for non-normal data, we used median (IQR) and the Wilcoxon rank-sum test. Categorical variables were summarized as percentages and compared with the chi-square test.

Initially, we evaluated potential multicollinearity among independent variables using the variance inflation factor (VIF).The VIF values for all variables were below the threshold of 5, with the exception of PT and INR. As INR is a standardized indicator of PT and has clearer clinical significance, only INR is retained in the model, and PT is excluded to avoid the influence of collinearity on the results. Subsequently, the proportional hazards (PH) assumption was tested using Schoenfeld residual tests for each variable in univariable Cox regression, followed by a global PH test for the preliminary multivariable Cox model incorporating all candidate covariates. Consistent with the complexity of critically ill populations with sepsis-induced coagulopathy, a considerable proportion of variables violated the PH assumption at the univariable level (p < 0.05), and the global test further confirmed that the standard Cox proportional hazards model was inappropriate for our data (p < 0.05). Due to these violations, we adopted a modified approach and constructed a Cox proportional hazards model with time-dependent covariates to ensure the robustness of our findings.

To identify potential confounders and minimize residual confounding, we utilized a change-in-estimate criterion-based variable selection approach, which aligns with epidemiological methodology and prioritizes the influence of confounders on the effect estimate of the exposure variable (ACAG). A 10% relative change threshold was predefined: a variable was deemed a clinically important confounder necessitating adjustment if its inclusion modified the hazard ratio (HR) of ACAG by >10%. Integrating this rigorous confounder control strategy, the final time-dependent Cox proportional hazards model incorporated ACAG (the primary exposure), SOFA, SAPS II, and lactate as covariates. Mediation analysis was not performed, as the relative changes in ACAG’s HR induced by vasopressor use, RRT, and IMV were all less than 10% and thus these variables were excluded from the model, indicating they did not meet the predefined threshold for being clinically meaningful mediators.

To compare the predictive performance of ACAG and AG, ACAG was dichotomized at the optimal cutoff of 17.7 mmol/L (determined via X-tile software), while AG was categorized using the clinically established threshold of 16 mmol/L. Kaplan-Meier (KM) curves were plotted to visualize inter-group survival separation, with significance assessed by the log-rank test. Receiver operating characteristic (ROC) curves were generated to evaluate their discriminatory abilities, and the DeLong test was used to compare the two area under the curves (AUCs).

Restricted cubic spline (RCS) analysis was performed to evaluate the predictive capacity and non-linear association between ACAG and in-hospital 30-day mortality risk.Subgroup analyses were conducted for ACAG by including variables such as age, gender, race, comorbidities (diabetes, myocardial infarction, congestive heart failure, and malignant cancer), and laboratory indices (BUN, total bilirubin, ALT, AST, ALP).All statistical analyses were carried out with the aid of X-tile (version 3.6.1), and R software (version 4.3.2). Statistical significance was defined as a p-value of less than 0.05 [18].

## Results

### Population and baseline characteristics

**Fig 1:**
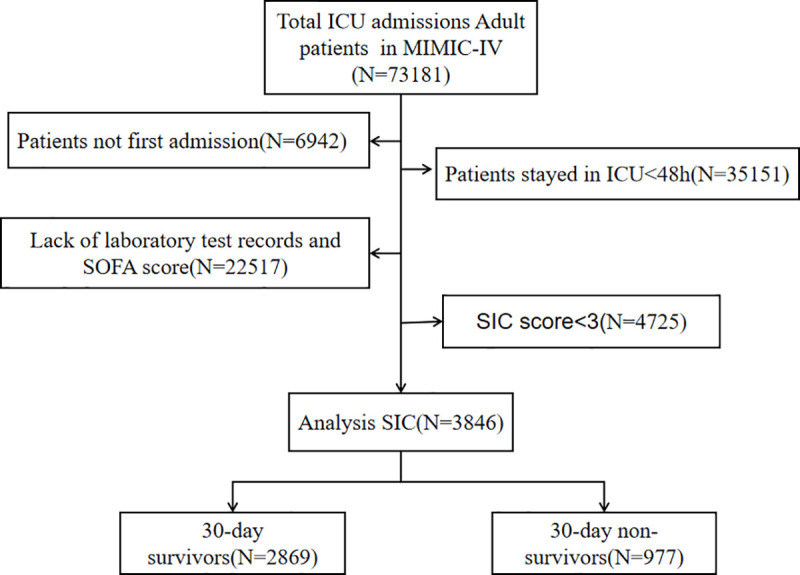
Study flowchart showing the inclusion and exclusion criteria.

A total of 3846 eligible patients were included in the analysis (Fig 1). The cohort comprised 1477 females (38.4%) with a mean age of 64.84 years (interquartile range 54.04-76.07). Non-survivors exhibited significantly higher values for age, SOFA score, SAPS II score, ACAG, WBC, BUN, INR, PT, PTT, ALT, ALP, AST, total bilirubin, lactate, and AG, as well as a greater need for life-supportive therapies. Conversely, survivors had higher levels of albumin. Additionally, survivors were generally younger and had a lower prevalence of myocardial infarction, congestive heart failure, renal disease, and malignancy compared to non-survivors (Table 1).

**Table 1:** Demographic and clinical characteristics of the study population. SIC score, Sepsis-induced coagulopathy; SASP II, Simplified Acute Physiology Score II.

Characteristics	30-day survivors(n = 2869)	30-day non-survivors(n = 977)	*P* value
Age(year)	63.86(52.83,74.89)	68.33（57.49,79.58)	<0.001
Gender(female %)	1081(37.7)	396(40.5)	0.113
RACE(%)			<0.001
White	1883（65.6）	564（57.7）	
African	282（9.8）	82（8.4）	
Others	704（24.5）	331（33.9）	
SOFA score	9.00(6.00,11.00)	11.00(8.00,14.00)	<0.001
SIC score(%)			0.004
4	1481(51.6)	469(48.0)	
5	627(21.9)	195(20.0)	
6	761(26.5)	313(32.0)	
SASP II	44.00(35.00,53.00)	53.00(44.00,64.00)	<0.001
ACAG(mmol/L)	14.65(11.65,18.40)	17.40(13.65,22.15)	<0.001
ACAG grade(%)	542(18.9)	332(34.0)	<0.001
Comorbidities(%)			
Diabetes	888(31.0)	309(31.6)	0.694
Liver disease	1079(37.6)	400(40.9)	0.064
Myocardial infarct	499(17.4)	212(21.7)	0.003
Congestive heart failure	958(33.4)	362(37.1)	0.037
COPD	742(25.9)	265(72.1)	0.439
Renal disease	711(24.8)	177(28.4)	0.027
Malignant cancer	436(15.2)	188(19.2)	0.003
Hypertension	1362(47.5)	469(48.0)	0.774
Laboratory parameters maximum			
WBC(*10^9/L)	14.10(9.50,20.30)	16.10(10.30,22.05)	<0.001
BUN(mg/dL)	30.00(19.00,47.00)	40.00(25.00,60.00)	<0.001
PT(s)	19.00(16.20,25.20)	22.00(17.45,31.75)	<0.001
PTT(s)	40.20(32.90,59.35)	45.90(35.10,70.50)	<0.001
ALT(U/L)	38.00(20.00,135.00)	46.00(22.00,169.50)	0.005
ALP(U/L)	87.00(61.00,133.00)	108.00(75.00,173.00)	<0.001
AST(U/L)	68.00(33.50,242.00)	93.00(42.00,326.00)	<0.001
Total bilirubin(mg/dL)	1.30(0.60,3.10)	1.40(0.70,4.20)	<0.001
Lactate(mmol/L)	2.90(1.70,5.10)	3.80(2.20,7.10)	<0.001
Anion gap(mmol/L)	18.00(15.00,21.00)	20.00(16.00,25.00)	<0.001
Laboratory parameters minimum			
Hemoglobin(g/dL)	8.90(7.50,10.50)	8.70(7.50,10.50)	0.328
Platelets(*10^9/L)	101.00(63.00,158.00)	99.00(56.00,166.00)	0.243
Albumin(g/dL)	2.90(2.40,3.30)	2.70(2.30,3.30)	<0.001
Glucose(mg/dL)	109.00(91.00,135.00)	108.00(84.00,139.00)	0.090
Ventilation(%)	2419(84.3)	869(88.9)	<0.001
RRT(%)	506(17.6)	302(30.9)	<0.001
Vasoactive drugs(%)	1954(68.1)	818(83.7)	<0.001

### Predictive performance of ACAG and AG for in-hospital 30-day mortality

**Fig 2:**
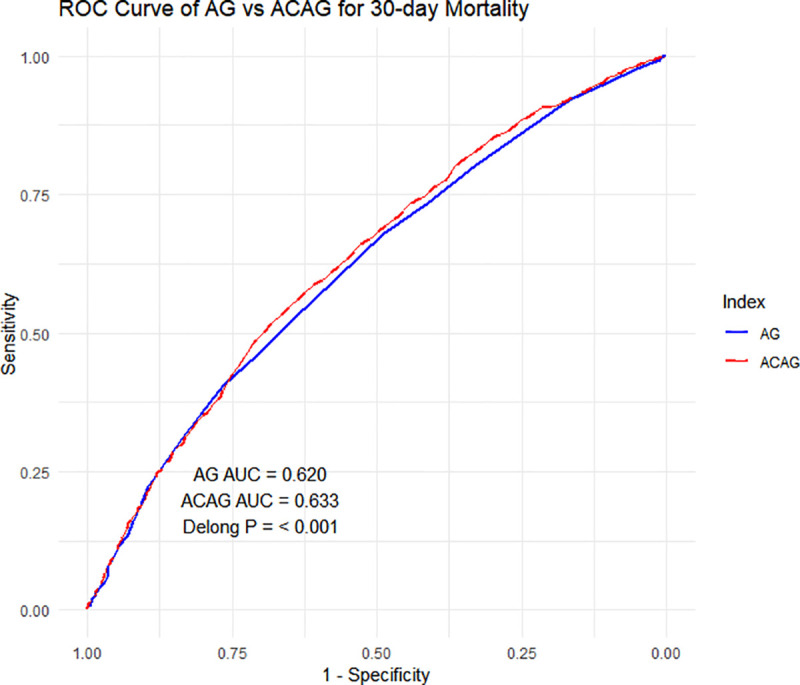
ROC of ACAG and AG.

**Fig 3:**
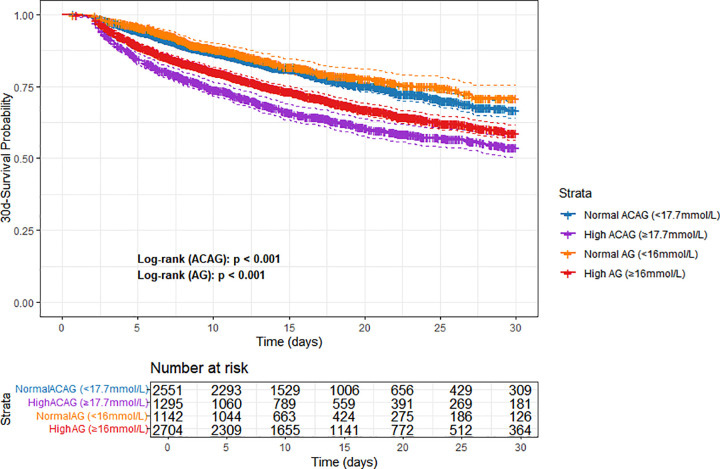
KM survival curve of ACAG and AG.

For in-hospital 30-day mortality prediction, ACAG demonstrated superior performance compared to AG: the AUC of ACAG (0.633) was higher than that of AG (0.620), with a statistically significant difference confirmed by the DeLong test (p < 0.001, Fig 2). Additionally, the log-rank test showed a larger chi-square value for ACAG (cutoff = 17.7 mmol/L, determined by X-tile software; 89.998) compared with AG (cutoff = 16 mmol/L; 40.681). Moreover, the Kaplan-Meier curves exhibited a clearer separation between ACAG subgroups (≥17.7 mmol/L vs. < 17.7 mmol/L) — stratified by the X-tile-derived threshold — than between AG subgroups (≥16 mmol/L vs. < 16 mmol/L), further supporting that ACAG has better prognostic discriminative ability than AG (Fig 3).

### ACAG and mortality: Cox proportional hazards model with time-dependent covariates

Table 2 presents HRs derived from the Cox proportional hazards model with time-dependent covariates. The overall goodness of fit of the model was assessed using the likelihood ratio test (χ² = 271.4, p < 0.001), Wald test (χ² = 295.4, p < 0.001), and Score (logrank) test (χ² = 310.4, p < 0.001), all of which indicated excellent model performance. In the primary effect analysis of ACAG (binary variable defined by a cutoff of 17.7 mmol/L determined by X-tile software; normal ACAG group = 1, high ACAG group = 2), patients in the high ACAG group had a 204.1% higher risk of in-hospital 30-day all-cause mortality compared to those in the normal ACAG group (HR = 3.041, 95% CI 1.883–4.912, p < 0.001). This effect was highly statistically significant, indicating that ACAG is a strong independent predictor of mortality in critically ill patients. Notably, the time-dependent effect of ACAG was statistically significant (p < 0.001): for each unit increase in log(t + 1), the prognostic effect of ACAG weakened by 32.0% (HR = 0.680, 95% CI 0.547–0.847). For lactate, the primary effect demonstrated that each 1-unit increase in lactate level was associated with a 7.2% increase in in-hospital 30-day mortality risk (HR = 1.073, 95% CI 1.035–1.112, p < 0.001). The time-dependent effect of lactate was also significant (p = 0.007): for each unit increase in log(t + 1), the effect of lactate weakened by 2.7% (HR = 0.973, 95% CI 0.954–0.993), reflecting a gradual attenuation of lactate’s prognostic impact over follow-up. For SOFA score, the primary effect analysis revealed no statistically significant association with in-hospital 30-day mortality (HR = 1.055, 95% CI 0.981–1.135, p = 0.149). Correspondingly, the time-dependent effect of SOFA score was also non-significant (p = 0.253; HR = 0.981, 95% CI 0.949–1.014), suggesting that SOFA score does not independently predict mortality in this cohort after adjusting for other covariates.

**Table 2:** Assessment of the impact of ACAG and covariates on 30-day in-hospital mortality using a Cox proportional hazards model with time-dependent covariates.

	HR for the main effect (95%CI)	HR for the main effect (95%CI)	P value for the time- dependent effect	HR for the time–dependent effect (95%CI)
ACAG group	<0.001	3.041(1.883-4.912)	<0.001	0.680(0.547-0.847)
Lactate	<0.001	1.073(1.035-1.112)	0.007	0.973(0.954-0.993)
SOFA	0.149	1.055(0.981-1.135)	0.253	0.981(0.949-1.014)
SAPS II	0.005	1.024(1.007-1.041)	0.845	0.999(0.992-1.007)

### Restricted cubic spline of ACAG levels versus in-hospital 30-day all-cause mortality

**Fig 4:**
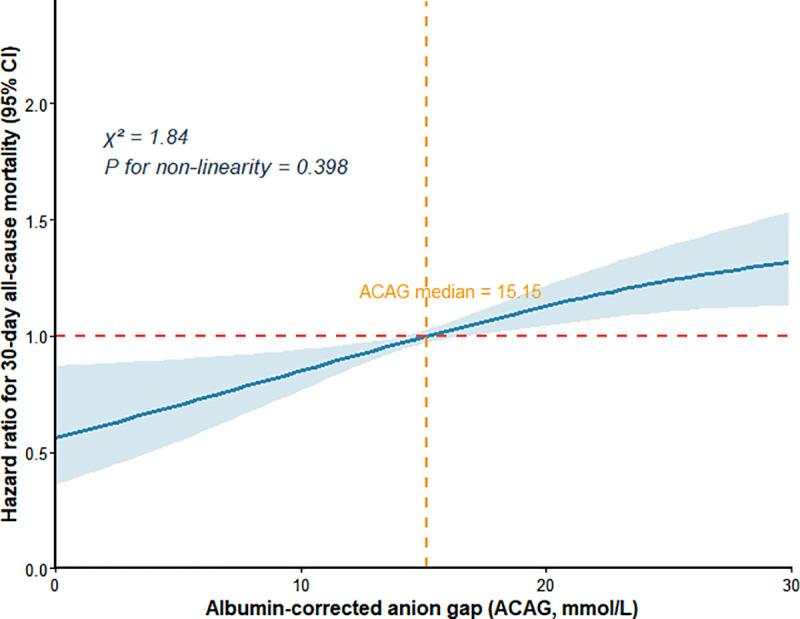
RCS analysis of ACAG.

RCS analysis with 3 knots revealed no statistically significant non-linear association between ACAG levels and in-hospital 30-day all-cause mortality (χ² = 1.84, *p* for non-linearity = 0.398). The RCS curve exhibited a steady increase in mortality risk with elevating ACAG levels (Fig 4), confirming ACAG as a reliable linear prognostic biomarker for in-hospital 30-day mortality in SIC patients.

### Subgroup analysis

**Fig 5:**
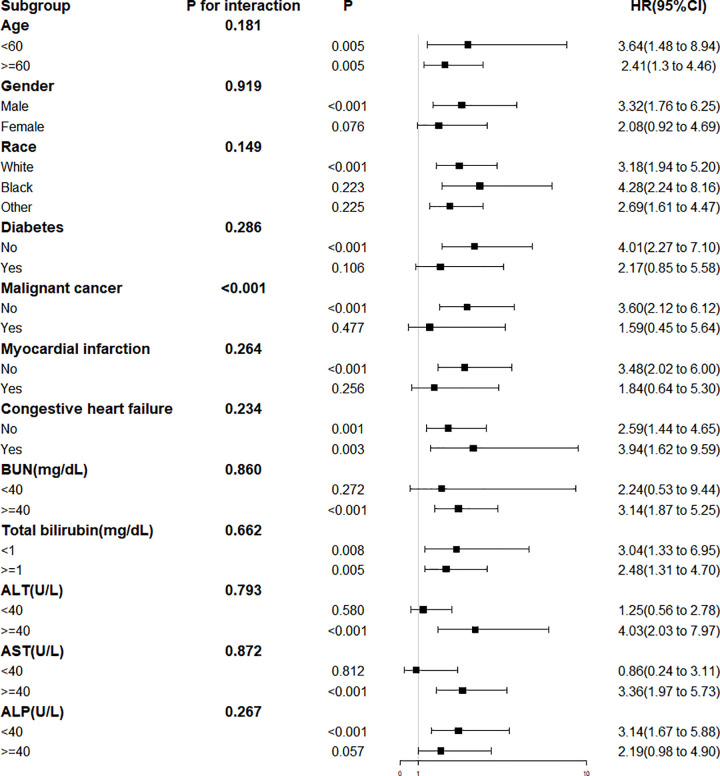
Subgroup analysis of ACAG.

Subgroup analysis showed that the association between elevated ACAG and mortality persisted across multiple strata (Fig 5). Regarding ethnicity, White patients (HR = 3.18, P < 0.001) and patients of other ethnicities (HR = 2.69, P = 0.225) exhibited relevant trends, while no association was observed in Black patients (HR = 4.28, P = 0.223). Significant associations were noted in patients without diabetes (HR = 4.01, P < 0.001) or malignant cancer (HR = 3.60, P < 0.001), but not in those with these comorbidities (diabetes: HR = 2.17, P = 0.106; malignant cancer: HR = 1.59, P = 0.477). Patients with ALT ≥ 40 U/L (HR = 4.03, P < 0.001) and AST ≥ 40 U/L (HR = 3.36, P < 0.001) had higher HRs. A significant interaction was only observed between ACAG and malignant cancer (P < 0.001), with no significant interactions noted for other factors (all P > 0.05).

### Enhanced predictive accuracy through combined models

**Fig 6:**
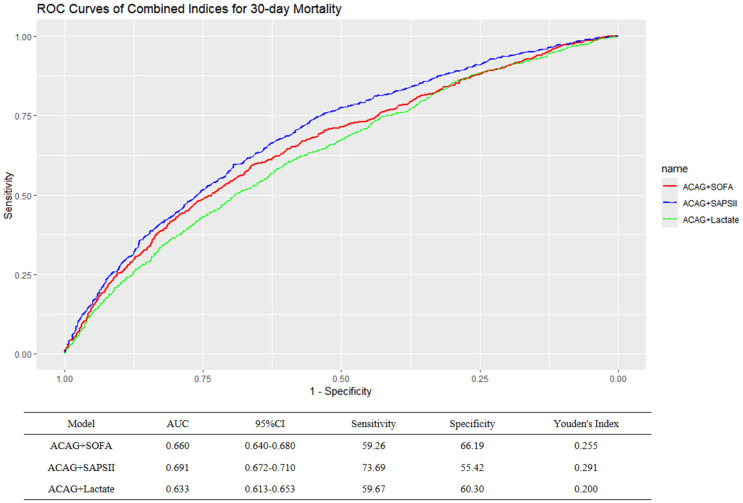
ROC curve of ACAG combined clinical indices.

Combining ACAG with clinical indices (SOFA, SAPS II, and lactate) significantly enhanced predictive accuracy for in-hospital 30-day mortality. As shown in Fig 6, the AUCs of the combined models (ACAG+SOFA: 0.660; ACAG+SAPS II: 0.691) were significantly greater than that of ACAG alone (AUC = 0.633, p < 0.001), further validating its utility in clinical risk stratification.

## Discussion

To the best of our knowledge, this study represents the first exploration of the association between ACAG and prognostic outcomes in SIC patients. The findings highlight a robust correlation between elevated ACAG levels and in-hospital 30-day all-cause mortality within this high-risk cohort. Notably, ACAG maintained a strong association with in-hospital 30-day all-cause mortality even after accounting for potential confounders, thereby underscoring its predictive capacity for adverse clinical outcomes.

Maintaining acid-base equilibrium is essential for normal physiological functions, and severe acid-base disturbances are often associated with an unfavorable prognosis. Relevant studies have demonstrated that sepsis can induce coagulopathy and acidosis, both of which, in turn, exacerbate the severity of sepsis [19–21]. This interplay among the three conditions forms a vicious cycle, collectively influencing disease progression and prognosis in patients. Prior research has shown that metabolic acidosis is prevalent among sepsis patients in the ICU and is linked to higher mortality rates [21]. The SIC score encompasses organ functions including coagulation, renal, hepatic, circulatory, and respiratory systems [14,15]. Owing to the immunosuppressive condition and complex alterations in organ function, these patients are at a heightened risk of developing multi-organ dysfunction and encountering elevated mortality rates [22]. Consequently, in this study, we concentrated on sepsis patients with coagulopathy, a particularly high-risk subgroup, to explore the relationship between metabolic acid load and clinical outcomes in this population.

AG represents the discrepancy between the concentrations of unmeasured plasma anions and cations and serves as a widely utilized indicator for evaluating acid-base balance in critically ill patients [23].

Elevated AG is often linked to augmented acid generation and reduced anion excretion [24]. In sepsis patients, hyperlactatemia is a prevalent acid-base disturbance [19]. Elevated lactate levels in sepsis patients are indicative of the severity of tissue hypoxia, circulatory dysfunction, metabolic derangements, and impaired coagulation function, and are associated with adverse clinical outcomes [25,26]. In addition, in patients with sepsis-associated coagulopathy, acute kidney injury (AKI) is a frequent complication and is associated with poor prognosis [27]. Kidney function impairment results in the build-up of diverse acids within the body, thereby causing an increase in serum AG levels. However, albumin is a crucial component of the AG, and hypoalbuminemia leads to reduced plasma albumin levels. In these situations, a normal AG might suggest that the decline in albumin levels has masked the elevation of plasma acids [28,29]. Considering the widespread occurrence of hypoalbuminemia among septic patients, the application of the ACAG may provide a more accurate assessment of acid load [30,31].

At present, research has revealed that in various diseases such as acute kidney injury, cardiovascular diseases, acute pancreatitis, and asthma, a high level of ACAG is associated with poor prognosis [7,11,32–34]. However, research regarding the correlation between ACAG and clinical outcomes in septic patients remains limited. Relevant studies have observed a significant correlation between ACAG and in-hospital mortality in septic patients. Compared with the AG and albumin, ACAG demonstrates superior predictive accuracy [35,36]. The same results were also obtained in our study. Notably, ACAG can be calculated instantaneously from routine admission laboratory parameters (sodium, chloride, bicarbonate, and albumin) without requiring additional blood draws, specialized assays, or incremental costs, rendering it a highly practical, bedside tool for early risk stratification. ACAG has great potential to become a clinically useful tool, as it reflects tissue perfusion and provides a more accurate measurement than direct AG value measurement. Furthermore, the ACAG is readily accessible and can be frequently utilized to direct clinical management during the assessment of patients’ conditions.

In addition, we observed a significant interaction between the presence of malignancy and ACAG. ACAG exhibited predictive value in septic patients with sepsis-induced coagulopathy who did not have malignancies. This indicates obvious differences in the predictive performance of ACAG depending on whether the patient has a malignancy. Malignancy may play a crucial role. A series of complex metabolic changes occur in the bodies of patients with malignancies. The rapid proliferation and high metabolic demands of tumor cells lead to the over-production of acidic substances in the body. Meanwhile, tumor-related treatments such as chemotherapy and radiotherapy may also impair renal function and affect the excretion of acidic substances, thereby resulting in an elevation of ACAG [37–39]. Moreover, patients with malignancies often suffer from issues such as malnutrition and immunodeficiency. These factors can further disrupt the acid-base balance, complicating the interpretation of ACAG and potentially reducing its predictive accuracy in this patient group. Previous studies have demonstrated that, compared with patients without malignancies, those with malignancies have a higher risk of infection, which significantly increases the mortality rate among patients with SIC [40]. Therefore, our study emphasizes the need to attach great importance to non-malignant SIC patients. In conclusion, our research indicates that ACAG is an easily accessible and cost-effective indicator, which holds significant predictive value for adverse outcomes in patients with sepsis-induced coagulopathy.

Nevertheless, our study has limitations. Firstly, although ACAG correlates with mortality and is easily obtained, the ROC, sensitivity, and HR values are relatively modest, suggesting that a single indicator is insufficient to predict complex SIC-related mortality; future research should incorporate ACAG into multi-marker prediction models. Secondly, the correction factor for hypoalbuminemia remains debated across literature (ranging from 2.0 to 2.5 mEq/L per 1 g/dL decrease in albumin), and the lack of universal standardization may limit the generalizability of specific ACAG cutoff values across institutions. Thirdly, AG (and consequently ACAG) can be influenced by non-metabolic factors such as severe dysnatremia, laboratory assay errors, or unmeasured cations, which may occasionally confound its interpretation as a pure marker of acid load. Fourthly, in the MIMIC-IV database, some patients lacked measured albumin or AG values, preventing ACAG calculation and potentially introducing selection bias. Fifthly, we only collected ACAG values within 24 hours of admission; due to changing patient conditions, treatments, and potential dilutional effects of large-volume crystalloid resuscitation (which may artificially lower albumin and alter electrolyte concentrations), we could not assess the dynamic trajectory of ACAG or its interaction with resuscitation protocols. Finally, as our study was conducted on a predominantly American population, whether these results can be generalized to other ethnic groups or healthcare settings remains uncertain, necessitating prospective validation in diverse cohorts.

## Conclusion

In the present study, we found that higher ACAG are associated with an increased risk of in-hospital 30-day mortality in SIC patients admitted to the ICU. Our results also suggest that ACAG is a reliable predictor of in-hospital 30-day mortality, exhibiting superior predictive performance compared to AG.

## Supporting information

S1 Fig Bootstrap-based calibration plot and Brier score analysis for the ACAG cutoff model. (TIF)

S2 Fig Decision curve analysis (DCA) of the categorical ACAG model for risk stratification. (TIF)
