# Supplementary figures and images for: Increased serum albumin corrected anion gap levels are associated with poor prognosis in sepsis-induced coagulopathy patients
Source: PLoS One. 2026 Apr 16;21(4):e0347039. doi: 10.1371/journal.pone.0347039 (PMC13086298; doi:10.1371/journal.pone.0347039)

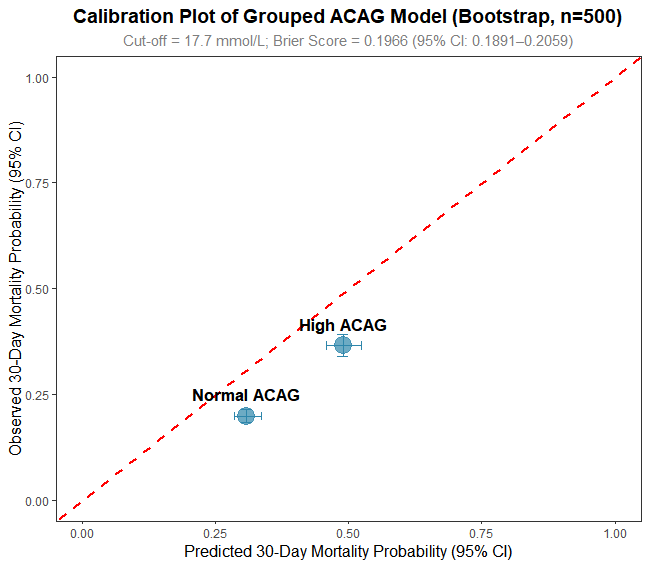

Supplement: S1 Fig — (TIF) [file pone.0347039.s001.tif]

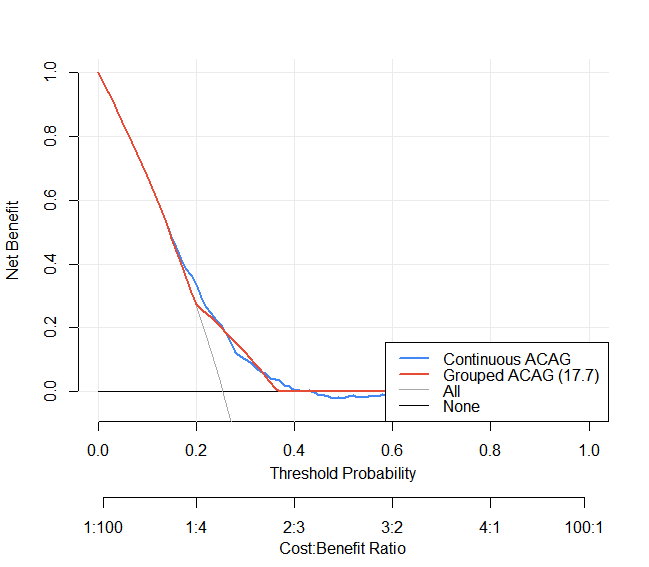

Supplement: S2 Fig — (TIF) [file pone.0347039.s002.tif]
